# Enhancing Performance of Organic Pollutant Degradation via Building Heterojunctions with ZnO Nanowires and Na Doped Conjugated 2,4,6-Triaminopyrimidin-g-C_3_N_4_

**DOI:** 10.3390/molecules29133240

**Published:** 2024-07-08

**Authors:** Ziyi Liu, Zixin Ruan, Xiaojie Yang, Yaqiong Huang, Jun Xing

**Affiliations:** 1 Hubei Key Laboratory of Radiation Chemistry and Functional Materials, School of Nuclear Technology and Chemistry & Biology, Hubei University of Science and Technology, Xianning 437100, China; 2 School of Biomedical Engineering and Imaging, Hubei University of Science and Technology, Xianning 437100, China

**Keywords:** ZnO nanowires, Na doped 2,4,6-triaminopyrimidine-g-C_3_N_4_, conjugate, photocatalytic performance, g-C_3_N_4_

## Abstract

Organic pollutants were one of the main sources of environmental pollutants. The degradation of organic pollutants through photocatalytic technology was one of the effective solutions. By preparing zinc oxide(ZnO) nanowires modified with sodium-doped conjugated 2,4,6-triaminopyrimidin-g-C_3_N_4_ (NaTCN) heterojunction (ZnO/NaTCN), the photocatalytic performance of NaTCN modified with different ratios of ZnO was systematically studied. The photocatalytic performance was studied through the degradation performance of methyl blue (MB) dye. The results showed that 22.5 wt% ZnO/NaTCN had the best degradation effect on MB dye. The degradation rate of MB reached 98.54% in 70 min. After three cycles, it shows good cycling stability (degradation rate is 96.99%) for dye degradation. It was found that there are two types of active species: ·OH and h^+^, of which h^+^ is the main active species produced by photocatalytic degradation of dyes. The excellent degradation performance was attributed to the fact that ZnO facilitated the extraction and transport of photogenerated carriers. The doping of sodium facilitated charge transfer. The NaTCN conjugated system promoted the extraction and transfer of photogenerated carriers. It provided guidance for designing efficient composite catalysts for use in other renewable energy fields.

## 1. Introduction

In the context of the national dual carbon strategy, photocatalytic technology can effectively solve energy and environmental problems, thereby achieving the goals of “peak carbon dioxide emissions” and “carbon neutrality” [1,2,3]. Photocatalytic degradation was considered a promising method for wastewater treatment due to its advantages of green environmental protection and no secondary pollution [4,5,6]. Photocatalytic degradation of pollutants was an oxidation-reduction reaction that had attracted widespread attention [7,8]. Phenol and its derivatives were common pollutants in industrial wastewater, typically generated in petrochemical processes, printing and dyeing industries, and food manufacturing [9,10].

Semiconductor photocatalysts could be divided into elemental semiconductor photocatalysts and composite semiconductor photocatalysts [11,12,13]. The good semiconductor photocatalyst had not only a narrow bandgap but also satisfied the absorption of longer visible light, and it had high charge separation and charge transfer rate, avoiding the recombination of internal and surface charges in semiconductors [14,15,16,17]. Researchers aimed to improve the recombination of photogenerated electrons and holes in photocatalysts by constructing heterojunction composite photocatalysts in order to compensate for the shortcomings of single semiconductor photocatalysts [18,19,20]. Graphitic carbon nitride(g-C_3_N_4_) was an environmentally friendly organic two-dimensional semiconductor photocatalytic material due to its reasonable band structure and narrow band gap [21,22,23]. However, as a photocatalytic material, g-C_3_N_4_ still faced key issues such as easy recombination of photogenerated carriers and insufficient surface catalytic ability, which seriously restricted its photocatalytic performance [24,25]. As a metal oxide semiconductor material, ZnO had advantages such as high electron mobility, rich morphology, easy regulation, high catalytic activity, strong chemical stability, nontoxicity, and low cost [26,27,28]. It had become a new star in the field of photocatalysts and had attracted the attention of scholars [29,30]. Dineshbabu et al. [31] used a simple co-precipitation method to prepare binary ZnO/g-C_3_N_4_, NiO/g-C_3_N_4_, and ZnO/NiO composite materials, as well as ternary ZnO/NiO/g-C_3_N_4_ composite materials. After 3 cycles, the degradation efficiency of ZnO/NiO/g-C_3_N_4_ composite material for tetracycline hydrochloride was 88.76%, indicating its advanced photocatalytic ability and high stability. Yu et al. [32] prepared sulfur-doped g-C_3_N_4_ by ball milling and one pot method. ZnO was prepared by calcining ZnO colloids. Preparation of S-doped ZnO@g-CN composite photocatalyst was prepared. The mechanism of S doping was studied. The main active free radicals generated by the undoped S photocatalyst sample were identified as ·OH radicals in the degradation process. The active free radicals generated by the S-doped photocatalyst sample were identified as ·O and ·OH radicals in the degradation process. Qiu et al. [33] prepared Ag-modified ZnO and g-C_3_N_4_ photocatalysts with different molar ratios using the electrostatic self-assembly method. The prepared Ag ZnO/g-C_3_N_4_ photocatalyst exhibits a unique 1D-0D-2D morphology and Z-type heterojunction. The prepared Ag-ZnO/g-C_3_N_4_ photocatalyst degraded 98% methylene blue within 30 min, which was superior to g-C_3_N_4_ (21% degradation within 30 min) and Ag-ZnO (84% degradation within 30 min). The excellent properties are mainly attributed to the Z-type heterostructure formed by silver nanowires and the localized surface plasmon resonance effect, which enables high-speed electron transfer between materials, expands the range of photoresponse, and reduces their impedance and photoluminescence. The composite of 1.0 wt% g-C_3_N_4_ and ZnO exhibited the best photocatalytic degradation efficiency. Doping elements could effectively improve photocatalytic activity, and the internal electron hole pair spatial transfer efficiency of g-C_3_N_4_ was enhanced, making the photocatalytic material more excellent. Introducing a conjugated system into the g-C_3_N_4_ structure was beneficial for regulating its own electronic structure, thereby changing the bandgap width, and enhancing photocatalytic activity due to the increased utilization of light [34,35]. The optimization of electronic structure was beneficial for enhancing the absorption of visible light, while the expansion of conjugated systems was beneficial for extending the carrier lifetime. Utilize the synergistic effect of the two to achieve efficient photocatalytic material preparation and application.

In this paper, melamine and triaminopyrimidine were used to prepare conjugated TCN systems through thermal polymerization. NaTCN was synthesized by thermal polymerization of melamine, sodium chloride, and triaminopyrimidine. ZnO nanowires was prepared by solid-state thermal decomposition. The series of ZnO/NaTCN composite materials was prepared by grinding method. The influence of the composition, structure, morphology, and other factors of the prepared materials on the catalytic degradation performance was systematically studied. The catalytic degradation performance of photocatalysts was evaluated by degrading MB (20 mg L^−1^) solution under visible light. The results showed that when the ZnO doping ratio was 22.5 wt%, the photocatalytic performance was optimal, and the degradation rate of MB (20 mg L^−1^) solution reached 98.54% within 70 min. After three cycles, the optimal photocatalytic activity of 22.55 wt% ZnO/NaTCN, the degradation efficiency did not fluctuate significantly, and it also showed good cycling stability for dye degradation. The efficiency improvement of the surface modification pathway of g-C_3_N_4_ by alkali metal ions can not only be effectively used for solar hydrogen production but also for promoting the photocatalytic degradation of pollutants. There is a strong ion dipole interaction between cations and the surface of g-C_3_N_4_. The Na^+^ cations in the solution are captured by negatively charged nitrogen atoms in g-C_3_N_4_, forming a surface coordination structure, which can improve the separation and transport characteristics of charge carriers in the material. Therefore, the photocatalytic activity of g-C_3_N_4_ has been significantly improved. Through active substance capture experiments to study the photocatalytic mechanism, it was found that there are two types of active species: OH and h^+^, of which h^+^ was the main active species produced by photocatalytic degradation of dyes. OH is a secondary active species in the catalytic degradation of MB by 22.5 wt% ZnO/NaTCN. It provided a new approach for the design and construction of g-C_3_N_4_ conjugated systems and the application of element doping in organic pollutants, catalysis, and energy fields.

## 2. Results and Discussion

### 2.1. Morphological and Structural Characterization

**Figure 1:**
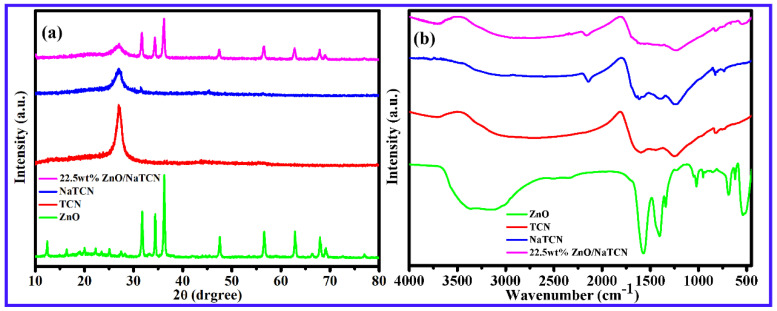
(**a**) XRD patterns of ZnO, TCN, NaTCN, 22.5 wt% ZnO/NaTCN; (**b**) infrared characterization of ZnO, TCN, NaTCN, 22.5 wt% ZnO/NaTCN.

As shown in Figure 1a, the samples of ZnO, TCN, NaTCN, and 22.5 wt% ZnO/NaTCN were characterized by FTIR and XRD. The XRD pattern showed that both TCN and NaTCN had a distinct diffraction peak located at 27.1°, representing the characteristic peak of graphite-like layered structure, corresponding to the interlayer stacking of aromatic compounds. It indicated that the doping of Na did not alter the crystal phase composition of TCN. The characteristic diffraction peaks of ZnO NWs at 31.72°, 34.34°, 36.12°, 47.63°, 56.64°, 62.92°, 66.37°, 67.94°, 69.08°, 72.65°, and 77.04° correspond to (100), (002), (101), (102), (110), (103), (200), (112), (201), (004), and (202) crystal planes (JCPDS NO. 99-0111), respectively. The results indicated that the prepared ZnO was wurtzite type. The XRD diffraction peak of 22.5 wt% ZnO/NaTCN composite material had the characteristic peaks of both NaTCN and ZnO, and no new diffraction peaks appear. The composite material was composed with ZnO and NaTCN, which fully proved the successful combination of the two and also indicated that the prepared sample had good crystallinity. As shown in Figure 1b, perform infrared characterization on the prepared ZnO, TCN, NaTCN, and 22.5 wt% ZnO/NaTCN. The samples of TCN and NaTCN exhibited several strong absorption peaks in the 1200–1650 cm^−1^ region, with peaks located at 1243 cm^−1^, 1405 cm^−1^, and 1615 cm^−1^, respectively. These absorption peaks were typical structures of TCN heterocyclic compounds, corresponding to C-N and C=N stretching vibrations in aromatic carbon nitrogen heterocycles, indicating that the introduction of Na did not alter these chemical bond structures. Compared with TCN, NaTCN exhibited a new absorption peak at 2141 cm^−1^, which could be attributed to the stretching vibration absorption peak of the carbon–carbon triple bond. The 22.5 wt% ZnO/NaTCN exhibited a weak absorption peak at 547 cm^−1^, mainly caused by the symmetric stretching vibration of ZnO, indicating the presence of ZnO in the composite material. The FT-IR spectra of 22.5 wt% ZnO/NaTCN showed similar structures compared to the characteristic spectra of NaTCN and ZnO, indicating that the composite material retained a graphite-like structure.

**Figure 2:**
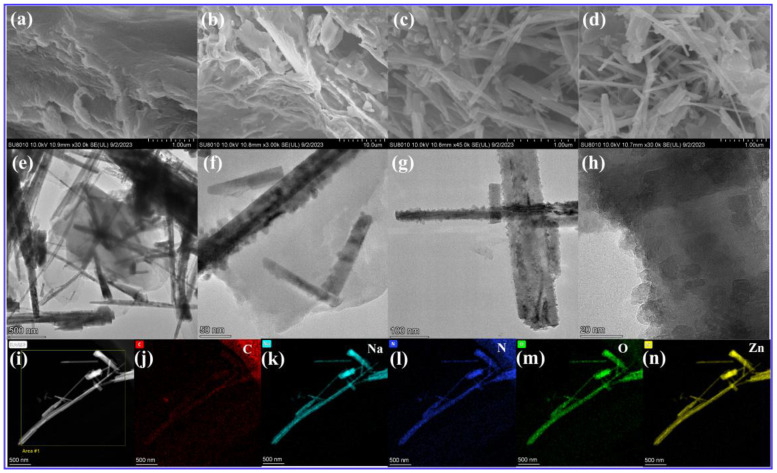
SEM images: (**a**) TCN; (**b**) NaTCN; (**c**) ZnO; (**d**) 22.5 wt% ZnO/NaTCN. TEM images: (**e**–**h**) 22.5 wt% ZnO/NaTCN. (**i**–**n**) TEM mapping images: 22.5 wt% ZnO/NaTCN.

Characterization of the microstructure of TCN, NaTCN, ZnO, and 22.5 wt% ZnO/NaTCN samples was carried out using SEM. Figure 2a showed that TCN exhibits a layered structure. Figure 2b showed the SEM image of NaTCN after introducing Na^+^, which clearly showed that the typical layered structure was still maintained, consistent with the previous XRD and FT-IR analysis results, and there were no components with Na^+^ crystal structure visible on the surface. This further indicated that metal Na^+^ interacted with TCN through ion coordination. Figure 2c showed the SEM images of ZnO were synthesized by direct solid-phase pyrolysis, with the prepared ZnO exhibiting a slender linear shape. Figure 2d showed the SEM images of 22.5 wt% ZnO/NaTCN composite material. The composite material had not only the layered structure of NaTCN, but also the linear-shaped ZnO distributed on the surface of NaTCN, which was conducive to the formation of heterojunctions and could enhance photocatalytic activity. To further analyze the microstructure of 22.5 wt% NaTCN/ZnO composite material was characterized by TEM, as shown in Figure 2e–h. The results showed that both NaTCN and ZnO nanowires with layered structure were present in the composite material, indicating that ZnO nanowires were uniformly distributed on NaTCN. The characterization results were consistent with XRD, FT-IR, and SEM. The elemental composition and distribution of the sample were determined using TEM-mapping, and Figure 2i–n showed C, Na, N, O, and Zn elements, all of which were uniformly present in the sample.

### 2.2. Evaluation of Photocatalytic Degradation Performance

Under visible light, MB was used as a simulated pollutant to investigate the photocatalytic performance of ZnO, TCN, NaTCN, and ZnO/NaTCN composite materials with different ratios. Firstly, 50 mg of ZnO and 50 mL of MB (20 mg L^−1^) solution were placed in a test tube. Subsequently, the test tube was placed in a photocatalytic reaction device and stirred in the dark for 30 min to achieve adsorption-desorption equilibrium between the photocatalyst and MB solution. Subsequently, turn on the Xenon lamp source and conduct photocatalytic degradation experiments under visible light irradiation. An amount of 2 mL of reaction solution was taken out at regular intervals until the reaction was completed. Finally, the supernatant was centrifuged at high speed with a 10,000 rpm centrifuge, and the absorbance values of the supernatant were measured using a UV-visible spectrophotometer at different reaction times. The degradation rate (%) was calculated using the following equation:

(1) $$Degradation\;Rate(\%)=\frac{C_{0}-C_{t}}{C_{t}}\times 100\%=\frac{A_{0}-A_{t}}{A_{0}}\times 100\%$$

Among them, C_t_ and C_0_ represented the corresponding concentrations of MB at time t and 0, respectively. A_0_ and A_t_ represent the corresponding absorbance of MB solution before and after illumination, respectively.

**Figure 3:**
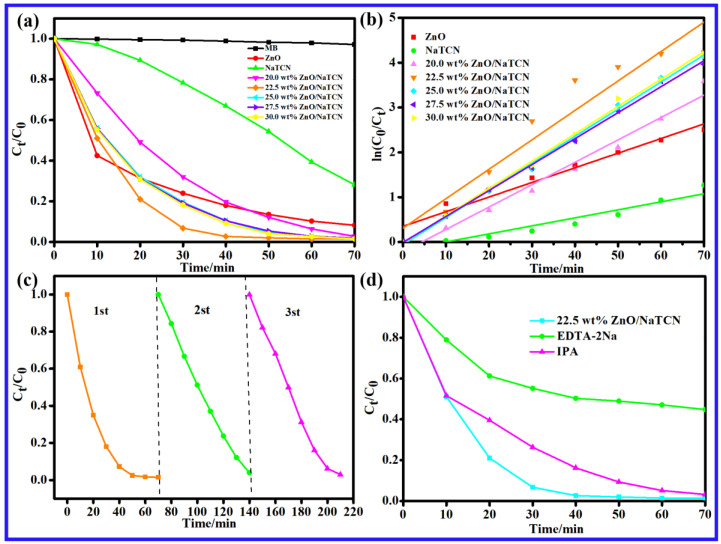
(**a**) Photocatalytic activity of photocatalytic degradation of MB under visible light; (**b**) The reaction kinetics curve of photocatalytic degradation of MB under visible light irradiation; (**c**) 22.5 wt% ZnO/NaTCN degradation MB cycle test diagram; (**d**) Capture experimental diagram of MB-related active substances degraded by 22.5 wt% ZnO/NaTCN.

The photocatalytic performance of ZnO/NaTCN composite materials with different doping ratios was generally superior to that of single ZnO and NaTCN, as shown in Figure 3a. When the doping ratio of ZnO was 22.5 wt%, the composite material formed at this time had the best performance in degrading MB, and the degradation rate of MB reached 98.54% within 70 min. The recombination between NaTCN and ZnO not only increased the contact area but also facilitated the formation of heterojunctions, enhancing the absorption capacity of visible light and suppressing the recombination ability of photogenerated carriers, thereby achieving the goal of improving photocatalytic performance. In order to further investigate the differences in MB photocatalytic degradation activity among different catalysts, first-order kinetic fitting was performed on the photocatalytic data. Figure 3b showed a linear fitting based on the formula −ln(C_t_/C_0_) = f(t). The corresponding kinetic rate constants are shown in Table 1, where C_t_ and C_0_ represent the concentration of MB in the solution after dark adsorption equilibrium and the corresponding concentration of MB at time t. Based on data analysis, the photocatalytic degradation of MB conforms to a first-order kinetic model, and the square value of the linear correlation coefficient (R^2^ > 0.90) indicates a good fit. The prepared composite catalyst exhibited a higher catalytic reaction rating than pure ZnO, with a maximum K value of 0.06566 for 22.5 wt% ZnO/NaTCN, where the doping ratio resulted in the best degradation effect, consistent with experimental results. In practical applications, catalysts not only had good photocatalytic performance but also had good cycling and stability.

Under visible light irradiation, cyclic stability was studied using 22.5 wt% ZnO/NaTCN as photocatalyst. Three cycles of experiments were conducted, as shown in Figure 3c. The experimental results showed that the degradation rates of MB for three times were 98.49%, 95.96%, and 96.99%, respectively. It can be seen that the 22.5 wt% ZnO/NaTCN composite material exhibited good stability and was easy to recycle and reuse. In order to fully study the mechanism of MB degradation by composite photocatalytic materials in Figure 3d. The active species was analyzed by involving in the photocatalytic degradation of MB by introducing capture agents corresponding to different active species into the reaction system. In this experiment, disodium ethylenediaminetetraacetic acid (EDTA-2Na) and isopropanol (IPA) were used as trapping agents for holes (h^+^) and hydroxyl radicals (·OH), respectively. As shown in Figure 3d, 22.5 wt% ZnO/NaTCN was analyzed by introducing capture agents corresponding to different active species in photocatalytic degradation of MB. The addition of EDTA-2Na as a capturing agent for h+ had a significant inhibitory effect on the degradation of MB, with the degradation rate reduced from 98.54% to 55.22%, indicating that h^+^ was the main active species in the photocatalytic degradation of MB by 22.5 wt% ZnO/NaTCN. The introduction of IPA as a capture agent for ·OH had no significant effect on the degradation of MB, and the degradation rate has decreased from 98.54% to 96.70%, indicating that ·OH was the secondary active species in the photocatalytic degradation of MB by 22.5 wt% ZnO/NaTCN. In summary, the active species for the catalytic degradation of MB by 22.5 wt% ZnO/NaTCN was mainly h^+^ under visible light. **·**OH is a secondary active species in the catalytic degradation of MB by 22.5 wt% ZnO/NaTCN.

**Figure 4:**
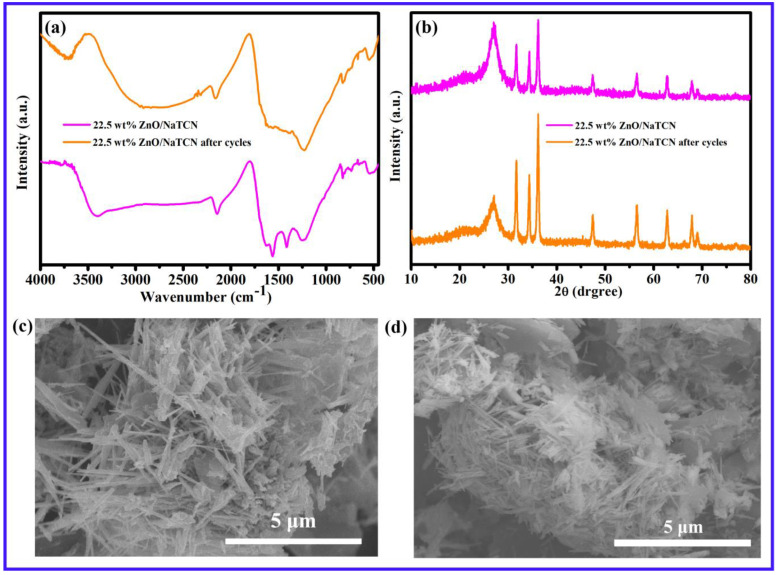
(**a**) Infrared characterization of 22.5 wt% ZnO/NaTCN, 22.5 wt% ZnO/NaTCN after cycles; (**b**) XRD patterns of 22.5 wt% ZnO/NaTCN, 22.5 wt% ZnO/NaTCN after cycles; (**c**) SEM images of 22.5 wt% ZnO/NaTCN; (**d**) SEM images of 22.5 wt% ZnO/NaTCN after cycles.

In order to study the structural stability and degradation mechanism of composite materials, FTIR, XRD, and SEM tests were conducted on the samples before and after cycling to investigate the material structure and functional groups in Figure 4. Through FTIR and XRD characterization, it can be seen that the corresponding functional groups have not changed, and the corresponding crystal structure has not changed in Figure 4a,b. Furthermore, it indicated that the prepared material did not adsorb dyes but rather degraded dyes through photocatalysis. Figure 4c,d showed SEM images of the samples before and after cyclic experiments. It could be observed that there was a slight agglomeration phenomenon after 22.5 wt% ZnO/NaTCN was recycled. It might be the main reason for the slight decrease in catalytic performance. However, the morphology of the catalyst did not change significantly, especially the linear structure of ZnO still uniformly distributed on the surface of NaTCN without detachment.

## 3. Methodology

### 3.1. Materials 

Zinc acetate dihydrate (CH3COO)_2_Zn·2H_2_O), Sodium chloride (NaCl), isopropanol (C_3_H_8_O, IPA), disodium edetate (C_10_H_14_Na_2_O_8_, EDTA-2Na),, and absolute ethanol (C_2_H_6_O) were purchased from Shanghai Sinopharm Chemical Reagent Co., Ltd. Shanghai, China. Melamine (C_3_H_6_N_6_), 2,4,6-triaminopyrimidine (C_4_H_7_N_5_) and Methylene blue (C_16_H_18_N_3_ClS, MB) were purchased from Shanghai Aladdin Biochemical Technology Co., Ltd. Shanghai, China. The chemical reagents used in this experiment were of analytical grade and were not further purified before use.

### 3.2. Preparation of TCN and NaTCN

**Figure 5:**
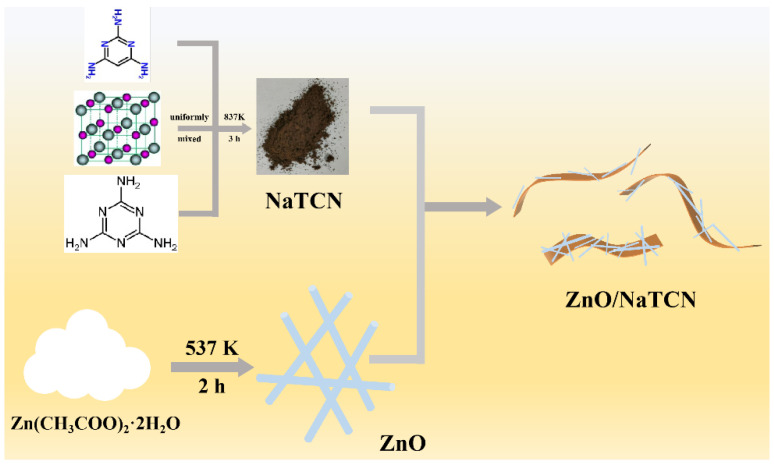
Schematic illustration of the fabrication of ZnO/NaTCN.

An amount of 2.0 g of melamine, 2.0 g of 2,4,6-triaminopyrimidine, and 6.0 mL of ethanol was grinded in a mortar for 10 min. After drying at 80 °C, the mixture was transferred to a crucible and annealed at 600 °C for 3 h in a muffle furnace in an air atmosphere with a heating rate of 3 °C min^−1^. After cooling to room temperature, the crude product was washed with boiling deionized water and ethanol twice. Finally, the product was obtained at 80 °C for 10 h. The preparation of Na-doped TCN was similar to TAP-CN: 2.0 g melamine, 4.0 g of sodium chloride, and 2.0 g 2,4,6-triaminopyrimidine were mixed grinding and calcination. The subsequent processing was the same as TCN preparation in Figure 5.

### 3.3. Preparation of ZnO Nanowires (ZnO NWs)

An amount of 7 g of Zn(CH_3_COO)_2_·2H_2_O was ground in agate mortar for 10 min and placed in the corundum crucible. The crucible was transferred to a muffle furnace and heated at a heating rate of 2 °C·min^−1^ to 300 °C for 2 h. Finally, ZnO NWs was obtained in Figure 5.

### 3.4. Preparation of ZnO NWs/Na-Doped TAP-CN (ZnO/NaTCN)

An amount of 0.1 g of NaTCN was added with the required mass of ZnO NWs (with doping amounts of 20 wt%, 22.5 wt%, 25 wt%, 27.5 wt%, and 30 wt%, respectively). The samples were obtained in Figure 5.

### 3.5. Characterization

Fourier transform infrared (FT-IR) spectra were acquired using a spectrometer (Nicolet 5700, Thermo Fisher Scientific, Waltham, MA, USA) to verify the presence and identify the specific vibration modes associated with functional groups. The sample’s crystal structure was determined using X-ray diffraction (XRD, Lab X XRD-6100, Shimazdu, Kyoto, Japan) with Cu-Kα radiation source. The measurements were taken with a working voltage of 40 kV and working current of 20 mA, and the scanning range spanned 2θ = 10~80°. The shapes and structures of the materials were examined using advanced imaging techniques, including field-emission scanning electron microscopy (FE-SEM, SU8220, Hitachi, Tokyo, Japan) and field emission transmission electron microscopy (FE-TEM, FEI Tecnai G2 F30, Hillsboro, OR, USA). Additionally, the ultraviolet-visible (UV-Vis) spectrophotometric method (TU-1950, Persee, Nanjing, China) was employed to study the dye degradation.

## 4. Conclusions

In this paper, TCN and NaTCN were prepared via thermal polymerization, in which Na was introducing a sodium source through an in situ doping method induced by a salt template. ZnO NWs was obtained by direct solid-state thermal decomposition. A series of ZnO NWs-doped NaTCN composite photocatalysts were prepared by depositing ZnO NWs on the surface of NaTCN. The prepared samples were characterized and studied for photocatalytic performance. The catalytic performance of photocatalysts was evaluated by studying MB degradation under simulated sunlight. The results showed that when the doping ratio was 22.5 wt%, the ZnO/NaTCN composite material had the best photocatalytic performance, with a degradation rate of 98.54% in MB (20 mg L^−1^) solution within 70 min. The study investigated the structural stability of 22.5 wt% ZnO/NaTCN material and conducted cyclic tests, demonstrating excellent degradation efficiency. The results showed that the catalyst exhibited excellent degradation efficiency and cyclic stability for dye degradation. This experiment provided ideas and a theoretical basis for conjugated systems and metal cation-doped conjugated systems.

## Figures and Tables

**Table 1:** Dynamics rate constants of degradation rate of MB by different catalysts.

	y = ln(C_0_/C_t_)	R^2^	Degradation Rate (%)
ZnO	y = 0.03264x + 0.34991	0.9472	91.81%
NaTCN	y = 0.0179x − 0.1772	0.90563	71.83%
20.0 wt% ZnO/NaTCN	y = 0.04995x − 0.21918	0.97607	97.22%
22.5 wt% ZnO/NaTCN	y = 0.06566x + 0.30978	0.91801	98.54%
25.0 wt% ZnO/NaTCN	y = 0.06045x − 0.05791	0.99636	98.42%
27.5 wt% ZnO/NaTCN	y = 0.05789x − 0.00893	0.99734	98.10%
30.0 wt% ZnO/NaTCN	y = 0.06097x − 0.02469	0.9965	98.48%

## Data Availability

The data presented in this study are available in article.
